# Training Convolutional Neural Networks on Simulated Photoplethysmography Data: Application to Bradycardia and Tachycardia Detection

**DOI:** 10.3389/fphys.2022.928098

**Published:** 2022-07-18

**Authors:** Andrius Sološenko, Birutė Paliakaitė, Vaidotas Marozas, Leif Sörnmo

**Affiliations:** ^1^ Biomedical Engineering Institute, Kaunas University of Technology, Kaunas, Lithuania; ^2^ Department of Electronics Engineering, Kaunas University of Technology, Kaunas, Lithuania; ^3^ Department of Biomedical Engineering, Lund University, Lund, Sweden

**Keywords:** photoplethysmogram, bradycardia, tachycardia, convolutional neural networks, detection, simulated signals

## Abstract

**Objective:** To develop a method for detection of bradycardia and ventricular tachycardia using the photoplethysmogram (PPG).

**Approach:** The detector is based on a dual-branch convolutional neural network (CNN), whose input is the scalograms of the continuous wavelet transform computed in 5-s segments. Training and validation of the CNN is accomplished using simulated PPG signals generated from RR interval series extracted from public ECG databases. Manually annotated real PPG signals from the PhysioNet/CinC 2015 Challenge Database are used for performance evaluation. The performance is compared to that of a pulse-based reference detector.

**Results:** The sensitivity/specificity were found to be 98.1%/97.9 and 76.6%/96.8% for the CNN-based detector, respectively, whereas the corresponding results for the pulse-based detector were 94.7%/99.8 and 67.1%/93.8%, respectively.

**Significance:** The proposed detector may be useful for continuous, long-term monitoring of bradycardia and tachycardia using wearable devices, e.g., wrist-worn devices, especially in situations where sensitivity is favored over specificity. The study demonstrates that simulated PPG signals are suitable for training and validation of a CNN.

## 1 Introduction

Continuous, long-term monitoring of atrial fibrillation using the photoplethysmogram (PPG) has received considerable attention in recent years, with early detection and prevention of serious health consequences, e.g., stroke, as main motivations Freedman et al. (2017); Pereira et al. (2020). Thanks to its simplicity, noninvasive PPG technology can be easily incorporated at a low cost in wearable digital devices for use in daily life. Among these devices, the wrist-worn is particularly attractive for continuous long-term monitoring Eerikäinen et al. (2020), relying on either traditional machine learning or deep learning for detection, e.g., Corino et al. (2017); Harju et al. (2018); Eerikäinen et al. (2018); Sološenko et al. (2019); Fallet et al. (2019); Selder et al. (2020); Väliaho et al. (2021). However, performance has so far only been established on short-term data due to the lack of public, annotated databases with long-term PPG recordings Eerikäinen et al. (2020).

While most research has focused on developing methods for PPG-based detection of atrial fibrillation, just a handful of studies has dealt with detection of other arrhythmias, notably premature atrial and/or ventricular beats Gil et al. (2013); Sološenko et al. (2015); Han et al. (2020) and bradycardia and ventricular tachycardia Bonomi et al. (2017). While neither bradycardia nor ventricular tachycardia are life-threatening arrhythmias, their extreme manifestations are known to be risk factors of serious conditions such as sudden cardiac death Harris and Lysitsas (2016). Using a wrist-worn device for continuous, long-term monitoring of bradycardia and tachycardia, valuable information may be acquired on initiating factors such as stress, medication, physical activity, and sleep Bonomi et al. (2017). Patients suffering from end-stage kidney disease undergoing hemodialysis treatment is a group of particular interest for such monitoring. Most studies point to that bradycardia, rather than tachycardia, is the pre-eminent pattern of serious arrhythmias and sudden cardiac death, with the highest incidence occurring during the interdialytic periods of conventional thrice-weekly hemodialysis Kalra et al. (2018); Foley et al. (2011); Boriani et al. (2015); Wong et al. (2015); Roy-Chaudhury et al. (2018). Continuous, long-term monitoring of extreme bradycardia in hemodialysis patients was recently established as an important procedure, accomplished using an implantable loop recorder Kalra et al. (2018). However, as an alternative, a wrist-worn device may be preferred as it offers the important advantages of low cost, low risk of infection, and avoidance of discomfort often experienced after insertion of the implantable loop recorder.

To detect bradycardia and tachycardia may seem like a simple problem solved by testing whether the heart rate is below/above a certain fixed limit for a certain minimum number of beats. However, such an approach tends to favor specificity over sensitivity Bonomi et al. (2017), without any means to alter the balance between the two performance measures. Irrespective of the approach taken to detection, the problem is made complicated by noise causing false detections. In addition, tachycardia with decreased hemodynamics is manifested in the PPG signal as much reduced or no pulsations, leading to missed beats when pulse-based detection is employed. These observations represent important incentives to explore new approaches to detection.

The present paper investigates the use of a dual-branch convolutional neural network (CNN) for PPG-based detection of bradycardia and tachycardia. The scalograms of successive signal segments, accounting for temporal and spectral information, constitute input to the network. To reduce the number of false alarms due to motion artifacts, a simple signal quality assessment is included in the detection process.

The main novelties of the present study are that, for the first time, a CNN is used to detect bradycardia and tachycardia, and that simulated PPG signals are employed for network training and validation. The performance of the CNN-based detector is also compared to that of a reference pulse-based detector.

The paper is organized as follows: Section 2 describes the datasets used for training, validation, and testing, Section 3 describes the proposed detector and the reference detector, Section 4 presents the results obtained on a clinical dataset, followed by a discussion in Section 5.

## 2 Datasets

Due to the lack of public PPG databases with annotated episodes of bradycardia and tachycardia, an unconventional approach is adopted in which simulated PPG signals are used for training and validation, whereas real, manually annotated PPG signals are used for testing. In the following, since the study focuses on ventricular tachycardia, tachycardia refers to ventricular tachycardia.

### 2.1 Datasets for Training and Validation

The simulator, originally developed to model PPG signals in paroxysmal atrial fibrillation using RR intervals alone as input Sološenko et al. (2017); Paliakaitė et al. (2019), is equally well-suited to model PPG signals with episodes of bradycardia or tachycardia; the simulator is freely available at Physionet Sološenko et al. (2021). The model signal is created by placing individual pulses according to the RR intervals so that a connected signal is formed, where each pulse is defined by a linear combination of a log-normal and two Gaussian waveforms. Stationary simulated noise, described in Sološenko et al. (2017), was added.

Different RR interval series with one episode of extreme bradycardia were created by concatenating three subseries of RR intervals, i.e., normal sinus rhythm, bradycardia, and normal sinus rhythm. The two subseries with normal sinus rhythm were randomly selected from the MIT–BIH Normal Sinus Rhythm Database Goldberger et al. (2000) so that 50–100 RR intervals appeared before the episode and 1–100 RR intervals after (the actual number of intervals before and after were selected randomly); in all subseries of sinus rhythm, the heart rate was above 60 beats per minute (bpm). In total, 147 RR interval subseries with bradycardia were selected from the PhysioNet/Computing in Cardiology (CinC) 2017 Challenge Database Clifford et al. (2017). Each series was approved by visual inspection to ensure that no aberrant RR intervals were included.

On the other hand, RR interval series with one episode, and in a few cases a handful of episodes, of tachycardia are contained in the Spontaneous Ventricular Tachyarrhythmia Database Goldberger et al. (2000). Since this database is not annotated, episode onset and end were determined manually, assuming a minimum episode length of three beats. In all recordings, tachycardia was surrounded by sinus rhythm, and, therefore, concatenation was superfluous. From the 135 recordings, a total of 94 RR interval series were selected with episodes having a heart rate of at least 120 bpm. The definitions of tachycardia and bradycardia are discussed in Section 5.

Table 1 summarizes the main characteristics of the dataset of simulated signals containing episodes of bradycardia and tachycardia.

**TABLE 1:** Main characteristics of the datasets used for training, validation, and testing.

Set	Characteristic	Bradycardia	Tachycardia
Training, validation	#RR interval series	147	94
	Total duration (h)	10	20
	#5-s segments	7,200	14,400
	#5-s segments with arrhythmia	1,092	437
	Min, median, max length (beats)	8, 23, 51	4, 14, 528
	Median heart rate (bpm)	36	164
Test set I	#recordings	15	39
	Total duration (min)	79	204
	#5-s segments	948	2,448
	#5-s segments with arrhythmia	52	64
	Min, median, max length (beats)	3, 4, 21	3, 6, 58
	Median heart rate (bpm)	38	142
Test set II	#recordings	15	29
	Total duration (min)	79	153
	#5-s segments	948	1836
	#5-s segments with arrhythmia	52	45
	Min, median, max length (beats)	3, 4, 21	3, 7, 58
	Median heart rate (bpm)	38	142

### 2.2 Dataset for Testing

**FIGURE 1:**
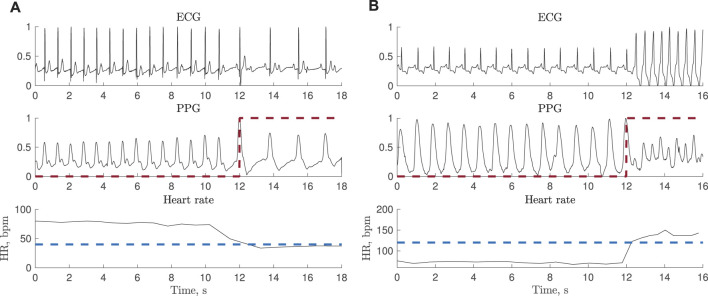
Synchronous ECG and PPG signals together with heart rate during **(A)** bradycardia ( <40 bpm) and **(B)** tachycardia ( >120 bpm). The ECG-based annotation is marked with a red dashed line. The signals are extracted from the PhysioNet/CinC Challenge 2015 Database.

The PhysioNet/CinC 2015 Challenge Database Clifford et al. (2015); Goldberger et al. (2000) is one of the very few PPG databases containing episodes of bradycardia and tachycardia and therefore used for testing. While each 5-min recording was originally assigned a rhythm label, indicating whether the recording contains bradycardia or tachycardia, episode onset and end was not annotated. Therefore, in the present study, episodes have been annotated using the simultaneously recorded ECG signals by relying on information on heart rate and beat morphology, assuming a minimum episode length of 3 beats. Figures 1A,B shows two excerpts from PPG and ECG recordings with bradycardia and tachycardia. In total, 15 recordings with bradycardia and 39 with tachycardia are used for testing, referred to as test set I; the Supplementary Table S1 lists all recordings. The total episode lengths of bradycardia and tachycardia are 79 and 204 min, respectively.

**FIGURE 2:**
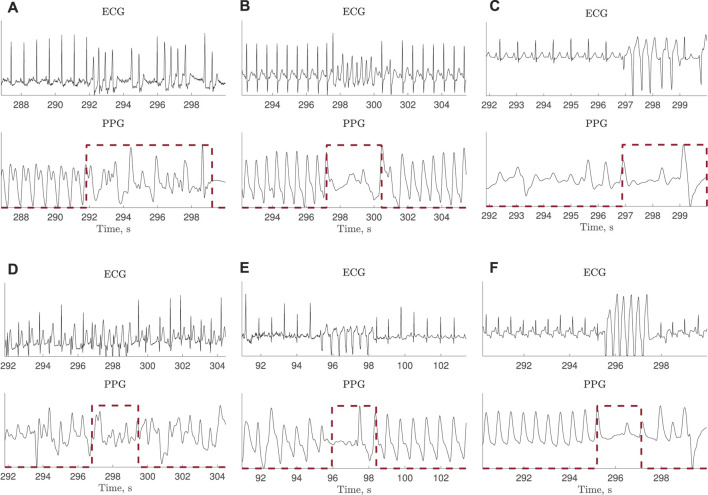
ECG and PPG signals with much reduced or no periodic pulsations during tachycardia. The ECG-based annotation is marked with a red dashed line. The signals are extracted from the PhysioNet/CinC Challenge 2015 Database.

Due to decreased hemodynamics during tachycardia, much reduced or no periodic pulsations were observed in 10 of the 39 recordings, illustrated in Figure 2. Therefore, a subset of test set I is defined excluding these 10 recordings, referred to as test set II.

Table 1 summarizes the main characteristics of the two test sets containing episodes of bradycardia and tachycardia.

## 3 Methods

**FIGURE 3:**
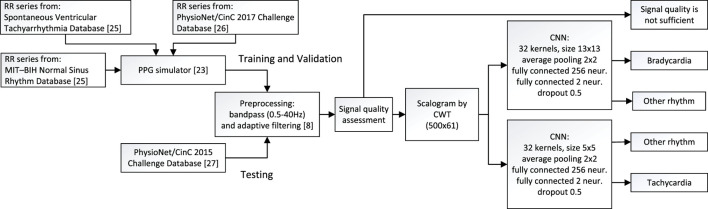
Block diagram of the method proposed for detection of bradycardia and tachycardia, including information on the datasets used for training, validation, and testing.

The method proposed for detecting bradycardia and tachycardia is composed of signal preprocessing and segmentation, signal quality assessment, and computation of the scalogram serving as input to the CNN-based detector. The block diagram in Figure 3 summarizes the detector structure as well as the datasets for training, validation and testing of the CNN-based detector.

### 3.1 Signal Preprocessing and Segmentation

The PPG signals, sampled at a rate of 100 Hz, are preprocessed using a bandpass filter with cut-off frequencies at 0.5 and 40 Hz. To further reduce the influence of baseline wander, an adaptive, normalized least mean squares filter is employed, with the reference input set to 1 Sološenko et al. (2019). Subsequent analysis is performed in non-overlapping 5-s segments.

### 3.2 Signal Quality Assessment

**FIGURE 4:**
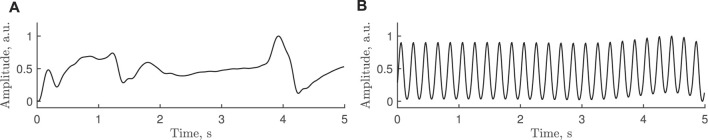
Examples of poor-quality PPG segments excluded after signal quality assessment, with the largest spectral peak at **(A)** 0.4 Hz and **(B)** 5 Hz, i.e., both frequencies outside the 0.6–3 Hz range.

To reduce the number of false alarms due to motion artifacts, signal quality is assessed by performing spectral analysis of the PPG signal. The location of the largest spectral peak within each 5-s segment is determined. If the peak is outside 0.6–3 Hz range, equivalent to 3–15 beats, which is a reasonable number of beats to occurs within a 5-s segment, the segment is assessed to be of poor quality and excluded from further analysis. Figure 4 shows examples of PPG segments excluded after signal quality assessment.

### 3.3 CNN-Based Detection

**FIGURE 5:**
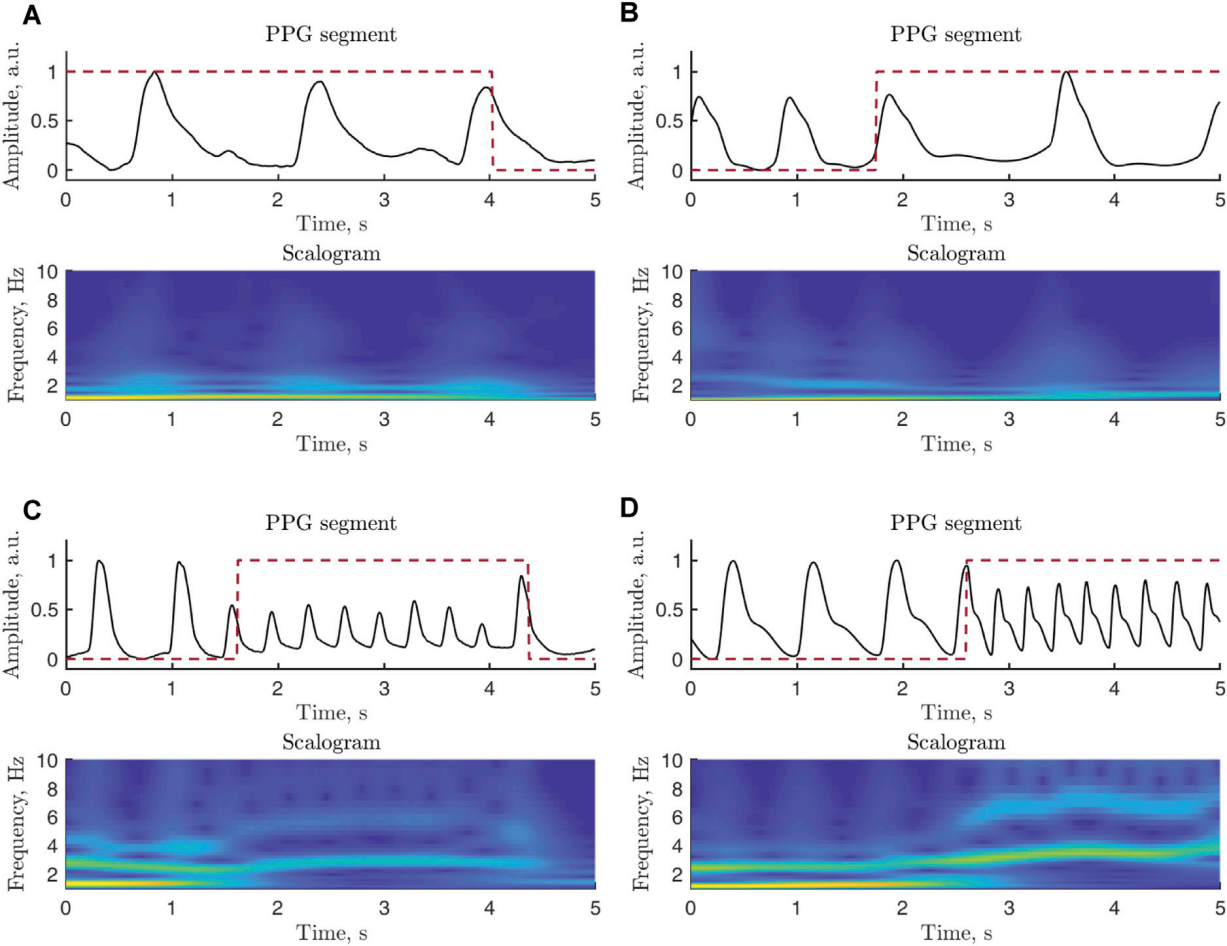
Examples of PPG signal segments and related scalograms: **(A)** real and **(B)** simulated signals in bradycardia, **(C)** real and **(D)** simulated signals in tachycardia. Since most of the power of a PPG signal is confined to lower frequencies, the vertical scale of the displayed scalograms is upper limited to 10 Hz. The annotation is marked with a red dashed line. The real signals are extracted from b124s and v837l of the Physionet/CinC 2017 Challenge Database, whereas the simulated signals are generated using A07531 of the Physionet/CinC 2017 Challenge Database and RRt3 of the Spontaneous Ventricular Tachyarrhythmia Database 1.0. The PPG signals have been normalized to [0,1] to facilitate comparison.

The continuous wavelet transform (CWT), offering good resolution in both time and frequency, is computed in each 5-s segment assessed to be of good quality. Using the generalized Morse wavelets, the resulting scalograms are treated as images with a size of 500, ×, 61 pixels, i.e., 500 samples and 61 scales. The minimum and maximum scales are determined by the distribution of the energy across the different scales. Figure 5 presents two examples of simulated and real PPG signals whose scalograms exhibit similar characteristics.

The detection of bradycardia and tachycardia relies on two CNNs (Supplementary Figure S1), where each arrhythmia is handled by its own particular model. Each model consists of two 2D convolutional layers with 32 kernels, where each kernel is followed by average pooling layers (size of 2 × 2 and a stride of 2) and two fully connected layers (input layer with 256 neurons and output layer with 2 neurons for segment classification). The kernel size of the two CNN models differ since bradycardia is composed of lower frequencies than tachycardia and therefore calls for a larger kernel size, here set to 13 × 13 (bradycardia) and 5 × 5 (tachycardia). The stride of the convolutional kernels is set to 1. All layers, except the output layer, are activated using rectified linear unit (ReLU) functions followed by a dropout rate of 0.5 to minimize overfitting; the output layer is softmax activated.

Before training the CNNs, the dataset of simulated signals is balanced by under-sampling the majority class, i.e., by randomly removing non-bradycardia (non-tachycardia) segments to match the number of bradycardia (tachycardia) segments. Then the dataset is split so that 70% is used for training and 30% for validation. The CNNs are trained using the Adam optimizer described in Kingma and Ba (2014) with a learning rate of 0.01. Training is stopped when the classification accuracy on the validation set stops improving.

Whenever the output of the bradycardia-trained CNN exceeds a certain threshold, the segment is classified as bradycardia, otherwise as other rhythm; the same applies to the output of the tachycardia-trained CNN except that another threshold is used. Both thresholds are chosen so that sensitivity is favored over specificity.

### 3.4 Reference Detector

For comparison, the pulse-based bradycardia and tachycardia detector described in Paliakaitė et al. (2021) was chosen. The PPG signal is bandpass filtered with cut-off frequencies at 0.5 and 6 Hz (instead of 40 Hz) to suppress high-frequency noise. The heart rate is obtained from the pulse-to-pulse intervals, where the occurrence times of the pulses are determined using a threshold-based detector similar to the one described in Aboy et al. (2005). The signal quality of each pulse is assessed by correlating it to a pulse template using the sample correlation coefficient. The quality is assessed as acceptable when the maximum correlation coefficient exceeds the threshold *η*_*c*_ = 0.6; for more details, see Sološenko et al. (2019); Paliakaitė et al. (2021). An episode of bradycardia is detected if the heart rate drops below 40 bpm for at least 3 high-quality beats, and an episode of tachycardia is detected if pulse rate exceeds 120 bpm for at least 3 high-quality beats. The output of the reference detector is divided into 5-s segments to facilitate a comparison of performance with the CNN-based detector.

### 3.5 Labeling of PPG Segments

Based on the annotation, each 5-s segment is labeled as either bradycardia, tachycardia, or other rhythm. Bradycardia is assigned if the episode lasts for at least 50% of the 5-s segment. Since tachycardia is characterized by higher frequencies, tachycardia is assigned if the episode lasts for at least 25% of the 5-s segment. The lower percentage reflects the obvious fact that more beats are contained in an episode of tachycardia than in an episode of bradycardia when both episodes have the same length in seconds.

### 3.6 Performance Measures

Detection performance is evaluated in terms of sensitivity and specificity by segmentwise comparison of the detector output to the labeling of the annotation described above. Sensitivity is defined by the number of correctly detected bradycardia (tachycardia) segments divided by the total number of bradycardia (tachycardia) segments, whereas specificity is defined by the number of correctly detected non-bradycardia (non-tachycardia) segments divided the total number of non-bradycardia (non-tachycardia) segments. These two measures are computed from the entire recordings, not just from segments assessed to be of good quality. The agreement between the CNN-based and reference detectors is evaluated in terms of Cohen’s kappa coefficient McHugh (2012).

## 4 Results

### 4.1 Performance as a Function of SNR

**FIGURE 6:**
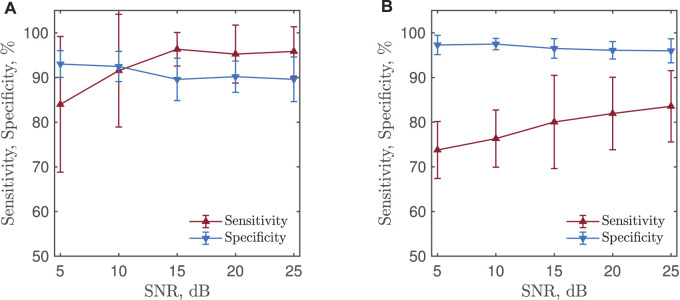
**(A)** Bradycardia and **(B)** tachycardia detection performance for a CNN trained with simulated PPGs at different SNRs. The results are based on test set II.

Figure 6 shows detection performance when the CNN was trained with simulated PPGs at different SNRs. For each SNR, 50 training sessions were performed and the average sensitivity ans specificity were obtained. Lowering the SNR of the training signals results in a decrease in sensitivity and an increase in specificity irrespective of whether bradycardia or tachycardia is detected. Since the best performance in terms of both sensitivity and specificity were obtained for noise-free PPGs when training the CNN, the CNN was trained with noise-free simulated PPGs before analyzing test sets I and II, see below.

### 4.2 Detection Performance on Test set I

**FIGURE 7:**
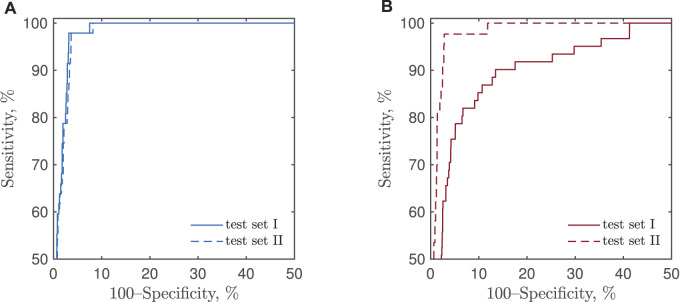
ROCs of CNN-based detection of **(A)** bradycardia and **(B)** tachycardia using test sets I and II.

Figure 7 presents the receiver operating characteristics (ROCs) of CNN-based detection of bradycardia and tachycardia, obtained by varying the two detection thresholds. No ROC is presented for the reference detector as its structure does not embrace a detection threshold.

Table 2 presents the performance of the CNN-based detector, using thresholds that put more emphasis on sensitivity, and the reference detector. Without signal quality assessment, the CNN-based detector offers higher sensitivity for both bradycardia and tachycardia and considerably higher specificity for tachycardia than does the reference detector. The exception is bradycardia specificity which is better for the reference detector.

**TABLE 2:** Performance and agreement of the CNN-based and reference detectors on test set I, without and with signal quality assessment (SQA).

Test set 1		Bradycardia		Tachycardia	
		No SQA	SQA	No SQA	SQA
CNN	Sensitivity,%	**98.1** [89.3, 100]	**98.1** [88.7, 100]	**79.7** [68.2, 88.5]	**76.6** [65.0, 86.1]
	Specificity,%	**96.7** [96.0, 97.2]	**97.9** [97.4, 98.4]	**95.6** [94.9, 96.3]	**96.6** [96.0, 97.2]
Reference	Sensitivity,%	**96.1** [89.0, 98.8]	**94.7** [87.2, 98.6]	**68.5** [57.1, 78.6]	**67.1** [55.6, 77.2]
	Specificity,%	**99.7** [99.5, 99.9]	**99.8** [99.6, 99.9]	**93.0** [92.1, 93.9]	**93.8** [92.9, 94.5]
	Cohen’s kappa	**0.42** [0.32, 0.51]	**0.49** [0.39, 0.59]	**0.39** [0.32, 0.46]	**0.39** [0.31, 0.46]

Square brackets indicate 95% confidence interval.

With signal quality assessment, the specificity increases for both detectors, although the increase is somewhat larger for CNN-based detection. The sensitivity decreases slightly for both detectors and arrhythmias, except for CNN-based bradycardia detection. This decrease is primarily due to the segments in which tachycardia is either contaminated with artifacts or the signal quality is low because of decreased cardiac output and perfusion leading to lack of periodic pulsations.

### 4.3 Detection Performance on Test set II

Table 3 presents the performance on test set II, i.e., test set I but excluding 10 problematic tachycardia recordings with much reduced or no periodic pulsations. As expected, the exclusion leads to improved sensitivity and specificity of both detectors. However, the increase in sensitivity of CNN-based detection is substantially larger than that of the reference detector. This is likely due to that the reference detector relies on pulse detection rather than on analysis of the whole 5-s PPG segment as does the CNN-based detector. For both detectors, signal quality assessment has only a minor effect on performance.

**TABLE 3:** Performance and agreement of the CNN-based and the reference detectors on test set II, without and with signal quality assessment (SQA).

Test set II		Bradycardia		Tachycardia	
		No SQA	SQA	No SQA	SQA
CNN	Sensitivity,%	**98.1** [88.6, 100]	**98.1** [89.1, 100]	**97.8** [87.7, 100]	**97.8** [87.6, 100]
	Specificity,%	**96.2** [95.4, 96.9]	**97.7** [97.1, 98.2]	**97.8** [97.2, 98.3]	**98.4** [97.9, 98.8]
Reference	Sensitivity,%	**96.1** [89.0, 98.8]	**94.7** [87.3, 98.6]	**74.5** [61.0, 85.3]	**72.6** [58.8, 83.9]
	Specificity,%	**99.7** [99.4, 99.9]	**99.7** [99.5, 99.9]	**96.8** [96.1, 97.4]	**97.6** [97.0, 98.2]
		Cohen’s kappa	**0.43** [0.33, 0.52]	**0.50** [0.40, 0.60]	**0.39** [0.29, 0.49]	**0.40** [0.29, 0.51]

Square brackets indicate 95% confidence interval.

**FIGURE 8:**
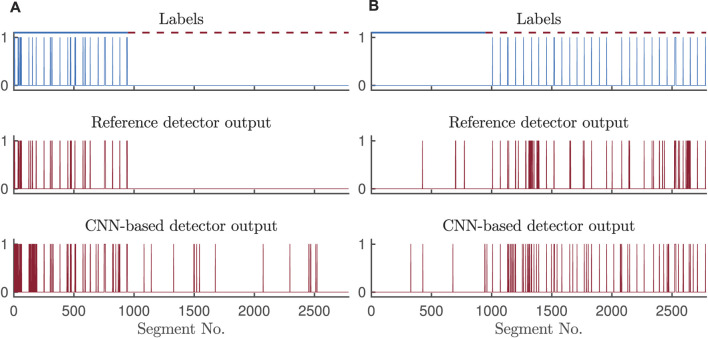
Outputs of the CNN-based and reference detectors for **(A)** bradycardia and **(B)** tachycardia detection on test set II, with signal quality assessment.

Figure 8 illustrates the outputs of the CNN-based and reference detectors together with correct labels. The Cohen’s kappa coefficient sheds some light on the disagreement between the detector outputs, mostly dictated by a small number of 5-s segments with arrhythmias in the two test sets, and different patterns of false alarms in either of the detectors.

## 5 Discussion

The present study shows that simulated PPG signals, based on real RR interval series, are practicable for training and validation of the CNN-based detector. Although the simulator offers the option to generate signals with realistic noise, noise-free signals were used for training and validation as this choice was found to produce better performance on the test set consisting of real PPG signals with occasional artifacts. However, if specificity is to be favored, noise should be added to the signals used for training and validation. On the other hand, randomly distributed noise episodes (i.e., nonstationary noise) may bias the training of the CNN-based detector, resulting in reduced performance.

A large bandwidth (0.5–40 Hz) of the bandpass filter was chosen so as to provide the CNN with rich training information. While a reduced bandwidth, e.g., 0.5–6 Hz used in the reference detector, may be motivated from a noise suppression standpoint, initial trials showed that the training and validation performance did not improve.

Thanks to the input segmentation, the CNN-based tachycardia detector is less sensitive to situations with reduced-amplitude pulsations than is the pulse-based reference detector since the scalogram carries additional information on tachycardia which helps to improve performance. This improvement is supported by the results in Table 2 which show that the sensitivity of the CNN-based tachycardia detector on test set I is superior to that of the reference detector, combined with better specificity of the CNN-based detector. The advantage of the CNN-based tachycardia detector becomes even more pronounced on test set II, see Table 3. Still, the CNN-based detector is susceptible to pulseless episodes as indicated by low sensitivity of tachycardia detection on test set I (see Table 2), which contained 10 recordings with much reduced or no periodic pulsations during tachycardia. Since these recordings are excluded from test set II, the sensitivity of the CNN-based tachycardia detector reported in Table 3 is considerably higher.

Pairs of pulses with a rate below 40 bpm or above 120 bpm are not considered an arrhythmia. However, the CNN-based detector may falsely detect bradycardia/tachycardia in segments where a single or a slow/fast pulse pair appears, resulting in lower specificity for bradycardia. Such behavior of the CNN may be the source of disagreement between the two bradycardia detectors, resulting in Cohen’s kappa values of 0.42–0.50. When detecting tachycardia, the CNN-based and reference detectors also exhibit different detection patterns as illustrated in Figure 8. Apparently, sensitivity of the reference detector is highly affected by the tachycardia-caused decrease in pulse amplitude resulting in missed beats. Even though the specificity for tachycardia detection is comparable, the sources of false alarms of the two detectors are different, and thus, the agreement in terms of Cohen’s kappa is low. Noise mimicking tachycardia misleads the reference pulse-based detector, whereas frequent premature beats might trick the CNN-based detector.

When reporting on detection performance, it is essential to state whether performance is computed using the annotations of all segments of the recordings or only the annotations of the segments which remain after signal quality assessment; the latter alternative tends to exaggerate the performance by ignoring false negatives corresponding to arrhythmia segments excluded due to poor quality Paliakaitė et al. (2021). In the present study, the performance measures are computed independently of segment exclusion since the annotations were determined from good-quality ECG signals, not from the PPG signals.

Several architectures of neural networks, including 1D CNNs, 2D CNNs, long short-term memory networks, and their combinations using either raw PPG signal or scalograms were investigated as a first step of the study. However, the best performance was achieved by using scalograms as input to the 2D CNNs. A rather basic CNN architecture was adopted in this study because its major objective was to demonstrate that a machine learning algorithm, trained on simulated data, can be employed to detect bradycardia and tachycardia in PPG signals. Thus, the comparison of different machine learning architectures and extensive testing of hyper-parameters were outside the scope of this study. Even though the proposed CNN-based detector is not complex, feasibility to implement and run it on a portable device should be investigated in the future.

A dual-branch CNN was selected for detection of tachycardia and bradycardia. Initial efforts showed that separate training of shallow network branches resulted in better performance than did one deep CNN. This result agrees with other studies proposing multi-branch structures of multi-class classifiers, e.g., Zhao et al. (2019). It has been argued that such structures are more robust in mitigating overfitting issues due to a small training dataset. Another advantage is that two parallel branches of the classifier allow parallel execution on separate kernels of the CPU or different threads in the software, resulting in reduced time to decision. Moreover, each branch of a dual-branch detector can function as an independent detector adapted to tachycardia or bradycardia detection.

In the present study, the output labels of the CNN branches were not merged, and the performance was reported separately for bradycardia and tachycardia detection. In no case was a segment labeled both tachycardia and bradycardia. However, in the extremely unlikely case when the same segment is labeled both bradycardia and tachycardia, the arrhythmia corresponding to the largest output should be selected.

CNN training with different segment lengths was not performed due to that bradycardia and tachycardia episodes are very brief. Segment labelling was defined so that bradycardia should occupy at least 50% of 5 s segment, while tachycardia should occupy at least 25% of 5 s segment. Therefore, using a different length, a segment containing bradycardia or tachycardia may not be labelled as an arrhythmia.

The prevailing clinical definition of bradycardia and tachycardia is a heart rate lower than 60 bpm and higher than 100 bpm, respectively, whereas no minimum duration is specified, see, e.g., Wagner and Strauss (2016). In the context of automated ECG analysis, various definitions can be found relating to the extreme manifestations of these two arrhythmias: extreme bradycardia is defined by a heart rate lower than 40 bpm with fewer than five beats within a period of 6 s, and extreme tachycardia is defined by a heart rate higher than 140 bpm with at least 18 beats within a period of 6.85 s Clifford et al. (2016). Yet another definition of extreme tachycardia can be found in Paliakaitė et al. (2021), replacing 18 with 5 beats, whereas the definition of extreme bradycardia remains unchanged; episodes has to be separated by at least 3 non-arrhythmic beats.

In the present study, the following definition is used to annotate the Spontaneous Ventricular Tachyarrhythmia Database and to evaluate the performance of the reference detector: bradycardia is defined by a heart rate lower than 40 bpm for at least 3 beats and tachycardia is defined by a heart rate higher than 120 bpm for at least 3 beats. One reason for using 120 bpm is due to that tachycardia slower than 140 bpm can still be life-threatening Roy-Chaudhury et al. (2018). It should be noted that none of these criteria apply to CNN-based detection as the scalogram serves as the basis for making informed decisions.

Tachycardia can have ventricular or supraventricular origin. In the present study, only ventricular tachycardia was investigated as it is more serious. Whether the PPG can be used to distinguish ventricular from supraventricular tachycardia remains to be demonstrated. Since the hemodynamics is more compromised by fast ventricular pacing, the amplitude of PPG pulses should in theory be less affected during supraventricular tachycardia. Still, the difference in PPG characteristics during ventricular and supraventricular tachycardia deserves to be investigated in future studies. The CNN-based detector may be trained to use such information, while the pulse-based reference detector is poorly suited for this purpose as it relies on heart rate only.

In the pioneering study on PPG-based detection of bradycardia and tachycardia Bonomi et al. (2017), only 3-min episodes and longer were detected. However, when the aim is to detect life-threatening episodes of extreme bradycardia and tachycardia, as is the goal of the present study, the minimum duration needs to be much shorter to ensure that an episode is composed of just a few beats. As a consequence, it is not meaningful to compare the present results to those in Bonomi et al. (2017). Of course, the intention to detect shorter arrhythmia episodes leads to increased number of false alarms or missed cases. However, since PPG-based detection is better suited for long-term monitoring outside the clinical setting, it could serve as a screening tool to initiate a clinical investigation of those at risk for life-threatening arrhythmias.

Using the arterial blood pressure signal as input, the problem of detecting bradycardia and tachycardia has been addressed by synthesis-by-analysis modeling Chou et al. (2019)—a technique closely related to the mixture models proposed in Liu et al. (2013); Sološenko et al. (2017); He et al. (2017). Such modeling results in a feature vector describing each pulse used for the classifier training [probabilistic neural network and random forest were investigated in Chou et al. (2019)]. This approach was found useful to the arterial blood pressure signal, however, it may be equally useful when applied to a PPG signal.

A limitation of the present study is the relatively small subset of short recordings from the PhysioNet/CinC 2015 Challenge Database used for the testing. Also, this subset does not include clinical data, and thus, it is unclear if some confounding factors can influence the performance of the CNN-based detector. However, to our knowledge, it is the only publicly available database with synchronous ECG and PPG signals with labeling of extreme bradycardia and tachycardia. Since the CNN-based detector was tested on recordings containing baseline sinus rhythm with episodes of bradycardia and tachycardia, it is unclear how the network generalizes to discriminate other arrythmias, e.g., atrial fibrillation. This issue deserves to be investigated in a future study.

## 6 Conclusion

A PPG-based bradycardia and tachycardia detector based on a dual-branch CNN is proposed, trained and validated on simulated PPG signals while tested on a dataset of real PPG signals. The results suggest that the proposed detector can be used for continuous, long-term monitoring, especially in situations where sensitivity is favored over specificity. In contrast to the reference detector, the CNN-based detector makes it possible to chose different operating points on the ROC. The study demonstrates that the use of simulated PPG signals is practicable for training and validation of a CNN.

## Data Availability

The original contributions presented in the study are included in the article/Supplementary Material, further inquiries can be directed to the corresponding author.
