# Supplementary material for: Training Convolutional Neural Networks on Simulated Photoplethysmography Data: Application to Bradycardia and Tachycardia Detection
Source: Front Physiol. 2022 Jul 18;13:928098. doi: 10.3389/fphys.2022.928098 (PMC9339964; doi:10.3389/fphys.2022.928098)
Supplement: Supplementary file 1 [file DataSheet1.PDF]

Supplementary Material

Table S1. Signals of the Physionet/CinC Challenge 2015 Database used for testing. The numbers correspond to the record names in the database.

#	Record	Rhythm	#	Record	Rhythm	#	Record	Rhythm
1	b227lr	B	19	v253lr	T	37	v714sr	T
2	b228sr	B	20	v254sr	T	38	v726sr	T
3	b229lr	B	21	v255lr	T	39	v729lr	T
4	b265lr	B	22	v275lr	T	40	v733lr	T
5	b299lr	B	23	v290sr	T	41	v748sr	T
6	b455lr	B	24	v309lr	T	42	v758sr	T
7	b456sr	B	25	v368sr	T	43	v769lr	T
8	b515lr	B	26	v369lr	T	44	v772sr	T
9	b516sr	B	27	v404sr	T	45	v773lr	T
10	b517lr	B	28	v471lr	T	46	v788sr	T
11	b656sr	B	29	v404sr	T	47	v797lr	T
12	b764sr	B	30	v628sr	T	48	v803lr	T
13	b794sr	B	31	v630sr	T	49	v806sr	T
14	b838sr	B	32	v632sr	T	50	v815lt	T
15	b839lr	B	33	v635lr	T	51	v818sr	T
16	v131lr	T	34	v648sr	T	52	v828sr	T
17	v132sr	T	35	v696sr	T	53	v831lr	T
18	v206sr	T	36	v701lr	T	54	v837lr	T

B/T stands for bradycardia/tachycardia.

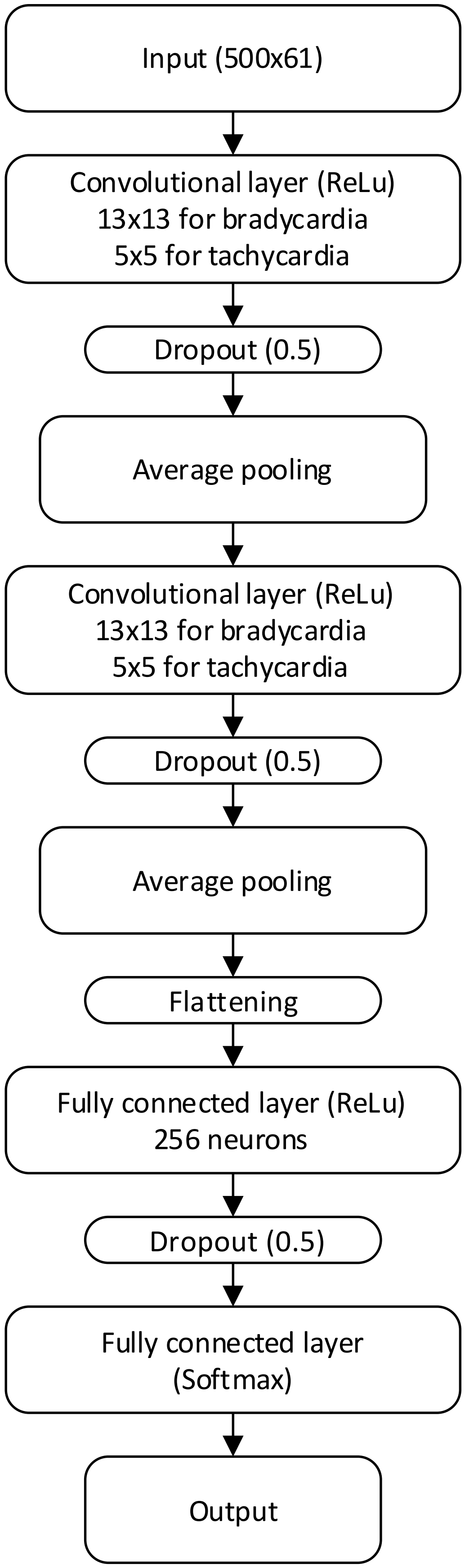

Figure S1: Structure of a CNN-based detector for bradycardia and tachycardia detection
